# Bovine mortality: the utility of two data sources for the provision of population-level surveillance intelligence

**DOI:** 10.3389/fvets.2024.1270329

**Published:** 2024-02-07

**Authors:** Jude I. Eze, Carla Correia-Gomes, George J. Gunn, Sue C. Tongue

**Affiliations:** ^1^Centre for Epidemiology and Planetary Health, Scotland’s Rural College (SRUC), Inverness, United Kingdom; ^2^Biomathematics and Statistics Scotland, Edinburgh, United Kingdom

**Keywords:** surveillance, cattle tracing scheme, bovine, mortality, fallen stock, aberration detection

## Abstract

**Introduction:**

The use of existing data to provide surveillance intelligence is widely advocated but often presents considerable challenges. Two data sources could be used as proxies for the mortality experienced by the Scottish cattle population: deaths recorded in the mandatory register [Cattle Tracing System (CTS)] and fallen stock collections by the National Fallen Stock Company (NSFCo) with a nationwide voluntary membership.

**Methods:**

Data for the period 2011–2016 were described and compared to establish their strengths and limitations. Similarities and differences in their temporal, seasonal and spatial patterns were examined overall, at postcode area level and for different age groups. Temporal aberration detection algorithms (TADA) were fitted.

**Results:**

Broadly, similar patterns were observed in the two datasets; however, there were some notable differences. The observed seasonal, annual and spatial patterns match expectations, given knowledge of Scottish cattle production systems. The registry data provide more comprehensive coverage of all areas of Scotland, while collections data provide a more comprehensive measure of the mortality experienced in 0–1-month-old calves.

**Discussion:**

Consequently, estimates of early calf mortality and their impact on the livestock sector made using CTS, or successor registers, will be under-estimates. This may apply to other registry-based systems. Fitted TADA detected points of deviations from expected norms some of which coincided in the two datasets; one with a known external event that caused increased mortality. We have demonstrated that both data sources do have the potential to be utilized to provide measures of mortality in the Scottish cattle population that could inform surveillance activities. While neither is perfect, they are complementary. Each has strengths and weaknesses, so ideally, a system where they are analyzed and interpreted in parallel would optimize the information obtained for surveillance purposes for epidemiologists, risk managers, animal health policy-makers and the wider livestock industry sector. This study provides a foundation on which to build an operational system. Further development will require improvements in the timeliness of data availability and further investment of resources.

## 1 Introduction

Most studies dealing with the mortality experienced by cattle populations have explored the potential use of statutory, or mandatory, registers in which deaths in the cattle population are recorded. Investigations have included use of these data for various purposes, including: as welfare indicators; to measure excess mortality in the presence of specific agents; to identify risk factors for and rates of mortality, and to develop systems to detect deviations from expected values (1–8). In Great Britain (GB), until recently, the British Cattle Movement Service (BCMS) had the statutory responsibility of recording all cattle deaths in England, Scotland and Wales. BCMS manages these data through the Cattle Tracing System (CTS) (9). While this data source may provide regular, comprehensive, temporal and spatially representative datasets, access to it in a timely fashion manner can be a challenge, especially for external parties. Consequently, the potential for use of such dataset to provide early warning systems for detecting aberrations in health events, while technically possible (10) may not be fully realized (3). Demonstration of this potential, through an examination of the utility of these data in retrospective analyses, may help to encourage the investment of both time and resources in the development necessary to move from applied research toward operational systems.

It is possible that there are alternative data sources that may be easier to access routinely. They may be complementary or may approach the challenge of measuring mortality from a different perspective, for example herd health records at farm level from benchmarking or herd health planning schemes. One data source that could provide a complementary indication of cattle mortality at a population level is fallen stock collections data. In two other countries, Spain and France, researchers have examined the utility of centralized fallen stock data for surveillance of health status of animal populations in different regions [(10, 11), respectively]. In Britain, the National Fallen Stock Company (NFSCo) collects such data as part of its business operations. A Community Interest Company (CIC), with voluntary membership, NFSCo provides access to competitive collection and disposal fallen stock services and prices to 44,000 farmers nationwide (12). NFSCo define fallen stock as “animals which were killed (euthanasia with or without definite diagnosis) or have died (including stillborn and unborn animals) on farm and which were not slaughtered for human consumption. This includes animals killed by routine culling as part of normal production arrangements” (13). It is therefore pertinent to also examine the utility of this alternative data source to determine if it too has the potential to contribute to surveillance intelligence.

The aim of this study was to evaluate these two sources of data, both related to deaths in cattle to determine their suitability and potential for use in an animal health surveillance system as a proxy for mortality experienced by the Scottish cattle population.

The objectives were to retrospectively (i) describe the mortality of Scottish cattle as provided by the two data sources- overall, by age-group and geographically; (ii) compare the temporal patterns and other characteristics of the fallen stock collections with that of the statutory death; (iii) investigate whether there is the potential for the use of aberration detection algorithms; (iv) compare the alarms generated by these algorithms in the two datasets and their subpopulations; and thus (v) identify any issues with the data that would present challenges in interpretation of any of the above objectives, or that may limit operational usage for surveillance activities.

## 2 Materials and methods

Cattle mortality data used in this study are for the period 2011–2016 inclusive, collected from two different sources: the Cattle Tracing System (CTS, hereafter mostly referred to as the registry data) and National Fallen Stock Company (NFSCo, hereafter mostly referred to as the fallen stock collections, or just collections data). The CTS database is managed by the British Cattle Management Services (BCMS) (9). It holds records of all births, on and off movements and deaths of cattle within the United Kingdom (UK). Cattle keepers are mandated to report the death of any cattle to BCMS (14). Data on all death events occurring in Scotland during the study period, excluding those at slaughterhouses, including animal unique identifiers, date of death, date of birth and farm/location of death were extracted from the collated and curated copy of the CTS database stored in the Scottish Government’s Centre of Expertise on Animal Disease Outbreaks (EPIC) data repository.^1^

NFSCo was established in keeping with the European Union Animal By-Product regulations (15) and UK Government’s guideline on fallen stock and dead animal safe disposal (16) which require that the disposal of dead farm animals is done through an approved, registered animal by-product premise. The NFSCo facilitates an efficient and competitive nationwide service for the collection and disposal of fallen farms animals and horses. This is accomplished by working with around 100 fallen stock collectors nationwide (12). Farmers can arrange for the collection of dead animals with any of the collectors in their postcode area. Farmers can also join as members of the company to facilitate collection and disposal. Membership is free and voluntary. The fallen stock collections data used was provided as aggregated data. They consisted of 217,255 records. Each contained information on the number of a stated livestock species (in this case bovines) of a specified age, that were collected in a given month/year, from a named Scottish postcode. They did not contain any record of production type.

The age at death recorded in the fallen stock data was provided in categories as follows: 0–1 month, 2–3 month, 4–6 month, 7–12 month, 13–23 month and 24+ month. For comparability, the calculated age at death in the registry data was categorized using the same age groups. These age categories are appropriate for investigating mortality at different time periods in a bovine lifetime. However, some data (28 out of 217,255 records) in the fallen stock collection dataset were not recorded as number of carcasses of a specified age group, but as “bags” with weight of the bags in units of 10 kgs. These were omitted from analysis as CTS data do not have contemporaneous data recorded in weight to compare with. Also, another 126 records were removed from the fallen stock data because their postcodes were in England. Consequently, 217,101 out of 217,255 (99.9%) records were used in the analyses. Each of the two datasets were aggregated to provide the number of deaths per month per year for the different age groups and postcode area.

Monthly time series plots of the number of deaths were used to compare the overall trend and seasonal patterns of the two datasets. The trends were captured by superimposing a smooth trend line on the monthly time series using locally weighted scatterplot smoothing (lowess) method. Differences in seasonal patterns and trends between age groups and geographic (postcode) areas of the two datasets were also examined. Seasonal patterns were represented as monthly boxplots, to show not only the seasonal pattern but also to reflect variations within year across months between the different age groups and the two datasets.

While the boxplots depict seasonal patterns, they do not give information on likely average contributions of each month (below or above the yearly average mortality) due to effects of production cycle, weather, and other factors. It is important to understand how the monthly effect differs between the registry and fallen stock collections data. To reflect this, we estimated monthly effects which indicate by how much the average number of deaths was influenced by a particular month. The effects were computed by first eliminating the trend by dividing each month’s mortality by the mean number of deaths in the same year. The resulting quotients were then averaged for each month across all the 6 years. To express this mathematically, if we denote the mortality in month i and year j as $y_{ij,}\;for\;i=1,2,3,\ldots .,12\;and\;j=2011,2012,..,2016$ . The seasonal or monthly effect $S_{i}$ is calculated as shown in Equation (1). These estimates were compared between the age groups in the two datasets. Note that the seasons are defined in this paper as winter (December, January, February), spring (March, April, May), summer (June, July, August) and autumn (September, October, November).

Correlation analyses between the monthly time series of the two datasets and by age groups were used to assess the strength of the linear relationship.


(1) $$S_{i}=\frac{1}{6}\overset{2016}{\underset{j=2011}{\sum }}\frac{y_{ij}}{{\bar{y}}_{j}}$$

In order to have a better understanding of the differences in spatial coverage of the two data sources, the percentage contribution of each postcode area to total mortality in each of the two datasets were represented on maps. Given that a greater proportion of the fallen stock data were calves aged 0–1 month old, it is of interest to see how the spatial distribution across the Scottish postcode areas differs from that of 0–1 month old category in the registry data. Consequently, the data were split into two: 0–1 month calves and the rest of the data. Differences in temporal patterns between these two groups were also examined.

The original Farrington algorithm (17) was used to explore the use of the two datasets for timely detection of aberrant counts of death. The choice of the algorithm is informed by its ease of use, can cope with smaller number of data in the reference and is commonly used in many established surveillance systems. It fits overdispersed Poisson generalized linear model with log link to the reference data and has the capacity to adjust for season and trend. Before fitting the algorithm, we validated the assumption of quasi-Poisson distribution by fitting a quasi-Poisson generalized linear model on the datasets as function of time and examined the behavior of the residuals.

If we denote $y_{i}$ as the number of deaths in month $t_{i}$ within a historical reference period and that $y_{i}$ is independently distributed with mean $\mu_{i}$ and an overdispersed variance $\theta \mu_{i}$ , then, the mean of the process can be modeled as a simple log-linear regression (Equation 2):


(2) $$\log\left(\mu_{i}\;\right)=\alpha +\beta t_{i}$$

Where α is the intercept and β is the slope of the trend line. Due to the assumption of overdispersion in the data, model estimates are obtained using quasi-likelihood approach. The algorithm implicitly adjusts for seasonal effect by estimating the current month’s expected number of deaths based on corresponding month in the reference period. The model is trained by first fitting it on the reference data and residuals examined for outbreaks. Outbreaks in the reference data are down-weighted by assigning very small weights to mortality with large residuals. The model is refitted on the adjusted reference values and the weighting is repeated. This procedure is iterative and continues until each predicted reference value is within the range of the reference values. This reduces the effect of past outbreaks on the estimate of current mortality. The trained model is then used to predict current mortality in the monitored period (Equation 3).

The predicted mean number of deaths in any given month $t_{0}\;$ in the current period that is being monitored is estimated as


(3) $${\hat{\mu }}_{0}=\exp\left(\hat{\alpha }+\hat{\beta }t_{0}\right)$$

To correct for skewness in the mean number of deaths, the algorithm use $\frac{2}{3}$ power transformation resulting to approximate symmetric distribution that enables the computation of confidence interval. The upper bound of the confidence interval above which current monthly mortality is declared an aberrant is given as (Equation 4):


(4) $$U={\hat{\mu }}_{0}{\left\{1+\frac{2}{3}z_{\alpha }{\hat{\mu }}_{0}-1{\left(\theta {\hat{\mu }}_{0}+\mathrm{var}({\hat{\mu }}_{0})\right)}^{1/2}\right\}}^{3/2}$$

with a desired confidence level and $z_{\alpha }$ is the (1- α ) percentile of the normal distribution [for more detailed account of this procedure, see (17)]. Given the short length of the monthly time series, the first 24 months (2011–2012) was used as the historic reference period. We show the results from the search of aberrations in the number of monthly deaths in year 2013 to 2016 using the original Farrington method. An upper bound U is calculated using the model predicted values, their variances and an alpha level of 0.01. An alarm is indicated when the observed number of deaths is higher than the predicted upper bound U . Aberrations detected in both datasets were compared to evaluate how close in time they occurred. We used similar set of parameters in the TADAs for ease of comparison of outputs from the two datasets. All data manipulation and analyses were conducted in R (18) and the TADAs were implemented using the surveillance package in R (19).

The outputs of the analyses were considered and interpreted in the light of expert knowledge of the underlying Scottish cattle population. This includes a diverse dairy industry and a beef industry, both with multiple production and management systems and a spatial distribution that is driven by geography, weather, land-use suitability and the human population distribution. A brief introduction is provided for the reader in Supplementary material.

## 3 Results

Within the period under study, a total of 450,903 deaths were registered in the registry (CTS) data for cattle in Scotland. Of these, 448,237 (99.4%) animals had information on date of birth. This compares with 445,676 individual animals collected as fallen stock in Scotland by NFSCo. The number in each age category varied between the datasets (Table 1). The fallen stock collections data provide a more comprehensive measure of the mortality experience in 0–1-month-old calves compared to the registrations in the registry data. About 55% of all fallen stock collections within the period of study were 0–1-month-old calves against 14% of the deaths registered in the registry data. Hence, the number of dead 0–1-month-old calves collected was 3.8 times the number recorded in the registry data. On average, 3,384 calves aged 0–1-month were collected each month from various farms compared to the 893 registered deaths in this age group each month. The picture is different for mortality in cattle aged more than 1 month. In the period of study, on average, about twice the number per age group were recorded in the registry data than were collected as fallen stock for the age groups over 1 month of age (Table 1).

**Table 1:** Proportion of fallen stock collected and registered deaths by age category in the two datasets Fallen Stock Collections (NFSCo) and register of mortality (CTS) between January 2011 and December 2016.

Age group	NFSCo *N* = 445,676 %	CTS *N* = 448,237 %	Ratio: (CTS/NFSCo)
Bovine 0–1 month	54.67	14.35	0.26
Bovine 2–3 month	6.27	11.30	1.81
Bovine 4–6 month	4.50	8.63	1.93
Bovine 7–12 month	4.34	9.92	2.30
Bovine 13–23 month	3.56	8.45	2.39
Bovine 24 month+	26.66	47.36	1.79
Total	100	100	1.01

### 3.1 Analyses of temporal patterns

**Figure 1:**
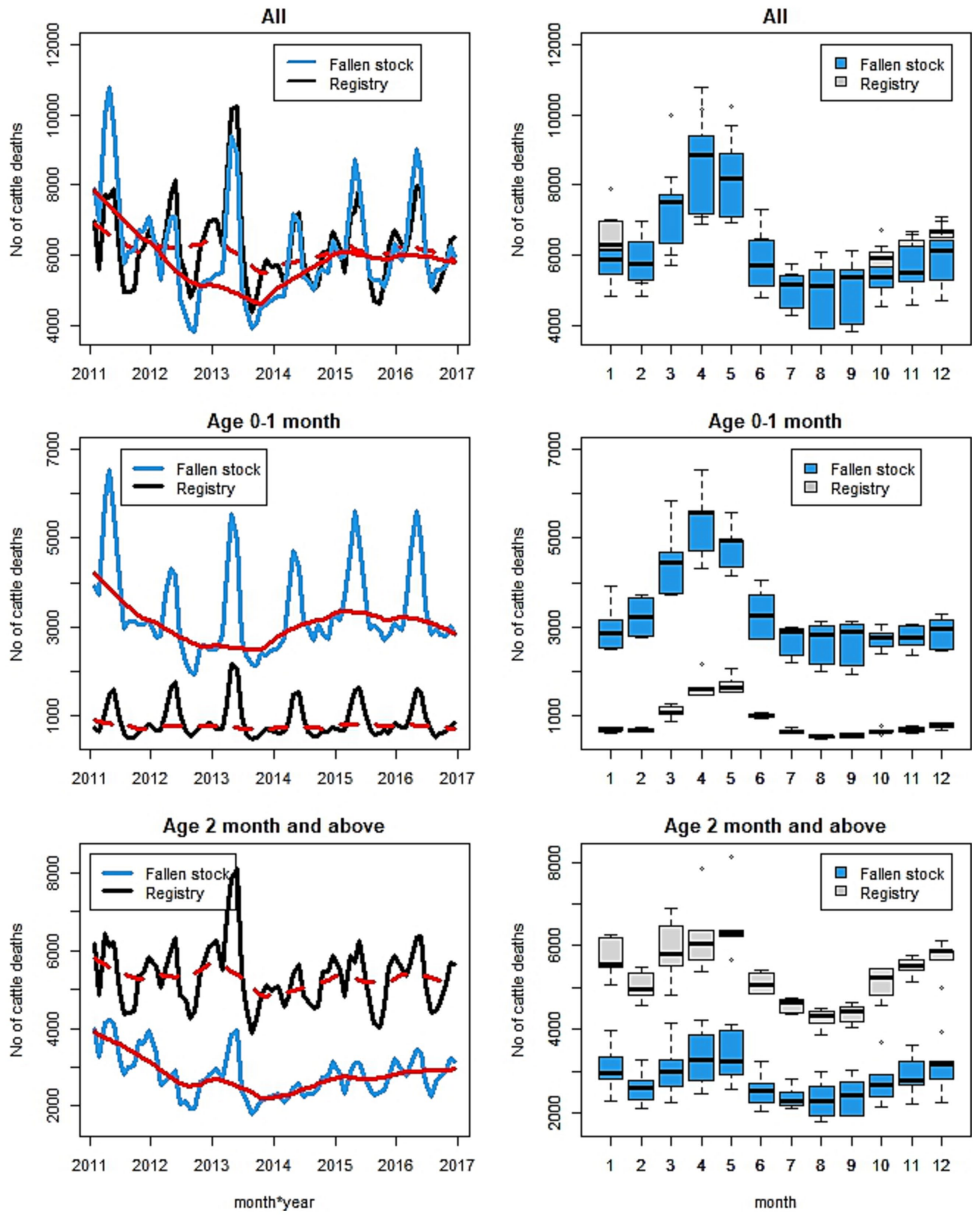
Plot of the monthly time series (first column) and seasonal patterns (second column) of number of fallen stock collected (in blue) and registered deaths (in black) for the overall (all-age) (1st row), calves aged 0–1 month only (2nd row), and the rest (excluding 0–1 month old) (3rd row). The red lines are the smooth trends—registry (dotted) and collections (solid).

The monthly number of cattle deaths in Scotland as represented by the registered deaths and the fallen stock collections has clear seasonal patterns and trends. Figure 1 shows the overall data series (first row) and the split of the data by calves aged 0–1 month (second row) versus others (third row). The first row shows the plot of the overall monthly time series and their seasonal patterns for the two datasets. The registry data are presented in black while the fallen stock collections data are in blue. The red lines are the smooth trends with that of the registry data shown as dotted red lines. Patterns are similar in the two datasets. Both time series show a decline until 2013 and a slow increase thereafter. The smooth trend is more pronounced in the fallen stock collections compared to the registry data. There are differences in the seasonal peaks across the years and these peaks seem to overlap in both datasets. The seasonal peak in April and May of 2013 are prominent. A better representation of the similarity in the seasonal patterns of the two monthly series is shown in the boxplots—the second graph in the first row. The boxplots suggest that peak mortality occurs in April and May (spring months), followed by the late autumn and early winter months, with the lowest number of deaths observed in the summer months. The estimates of monthly effect indicate an increased number of deaths of up to 1.4 times the yearly average number of deaths in spring (April) and about 20% reduction below the average yearly mortality in August (summer). A breakdown of the monthly effects by age groups suggest that effects differ depending on age (Supplementary Figure S2A).

**Figure 2:**
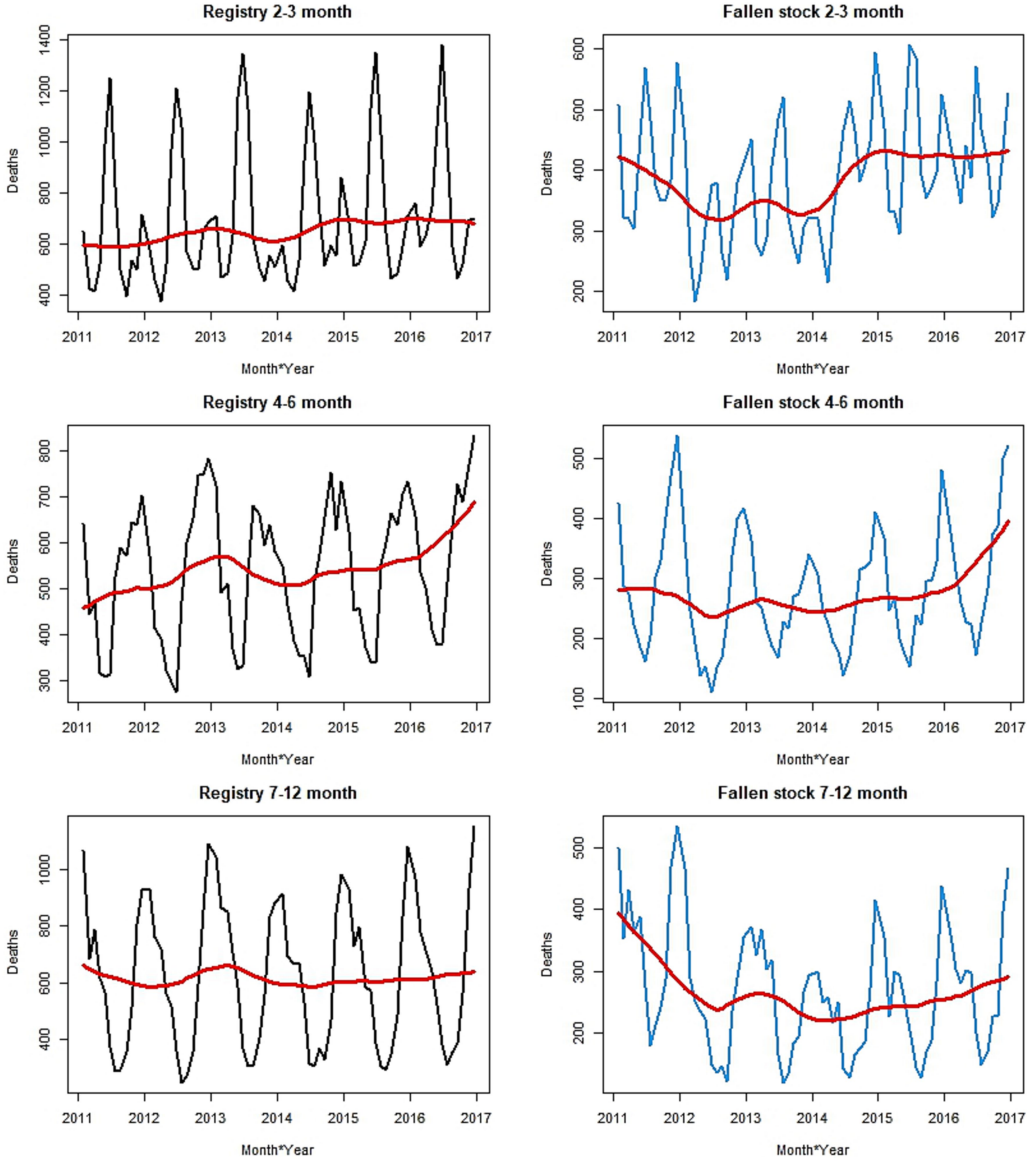
Monthly time series plots of mortality by age group, up to 12 months old, and by data sources. The smooth trend is shown in red.

**Figure 3:**
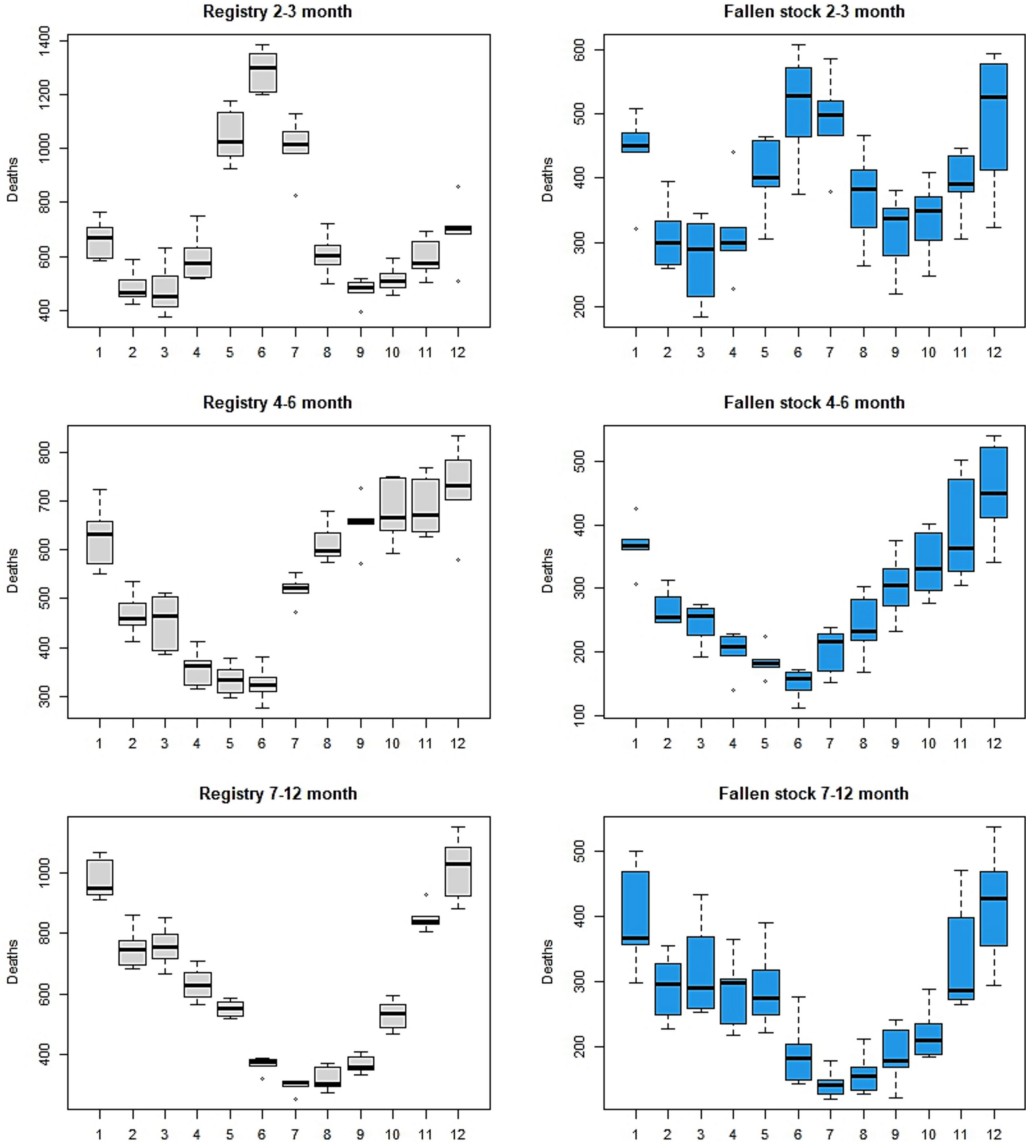
Seasonal patterns of mortality for each age group up to 12 months old, by the two data sources.

In the age groups up to a year of age, trends and seasonal patterns are different for different age groups (Figures 2, 3) in both datasets. Trends are well defined among the fallen stock age groups while those of the registry data show little or no change over the years. The exception is that of the 4–6-month-old calves, in which there was an increase over time. There appears to be a demarcation of seasonal patterns by age groups. The shape of seasonal patterns is similar in the two youngest aged (0–1 and 2–3 months olds) and also similar among the older age groups (4–6 and 7–12 months old). The drastic change in the seasonal patterns after 3 months of age and the shift in the seasonal peaks with increasing age is noteworthy in Figure 1 (second row) and Figure 3. The seasonal peaks changed from April in the 0–1 month old to June in the 2–3 month old in fallen stock data and from May to June in the registry data. The shift in peak with older age is even more prominent when we compare the 0–1 month group with 4–6 and 7–12 month old groups where peaks are in the late autumn and winter months. For the two age-groups over 12 months of age (13–23 months and over 24 months old), there are spring peaks and a late autumn-winter peak in the 13–23 month group that is most obvious in the registry dataset. Peak mortality in the over 24 months old is in spring (Supplementary Figure S2B). For all the age groups, the minimum number of deaths were observed in the summer and early autumn in both datasets. The differences in seasonal patterns among the age groups match expectations, given knowledge of the age groups and production cycles in the Scottish cattle sector (Figure 3 and Supplementary Figure S2B). Overall, more variations were observed on monthly basis in the fallen stock collections data compared to the statutory registry data.

### 3.2 Examining the extent of relationship between the two datasets

**Figure 4:**
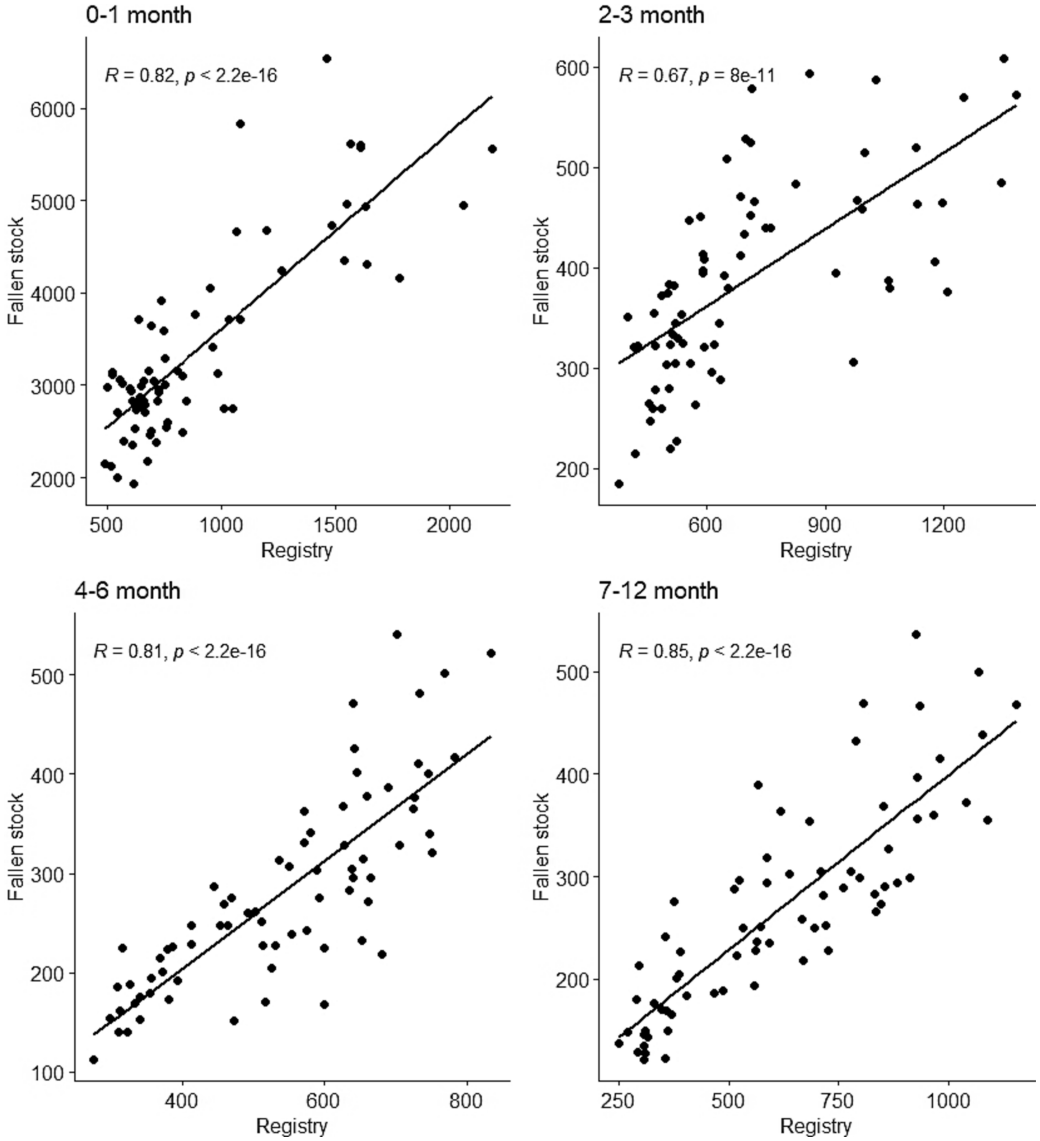
Scatter plots of the monthly time series of registry and fallen stock collections cattle data by age groups up to 1 year old. Moderate to strong positive correlation exist between the monthly time series of the age groups in two datasets.

The estimate of Pearson correlation coefficient indicates a significant and strong positive (0.79, *p* <0.001) correlation between the monthly time series of the two sets of data (Supplementary Figure S3). This investigation was extended to the monthly time series of the age groups. There was a strong positive correlation between corresponding age groups in the two datasets. Estimated correlation coefficients for cattle aged up to 12 months old range from 0.67 in the 2–3 month old group to 0.85 in the 7–12 month old group (Figure 4). While the over 24 month group also has a strong positive correlation 0.84, that of the 13–23 month age group is lower at 0.6 (Supplementary Figure S4).

### 3.3 Temporal aberration detection algorithm

**Figure 5:**
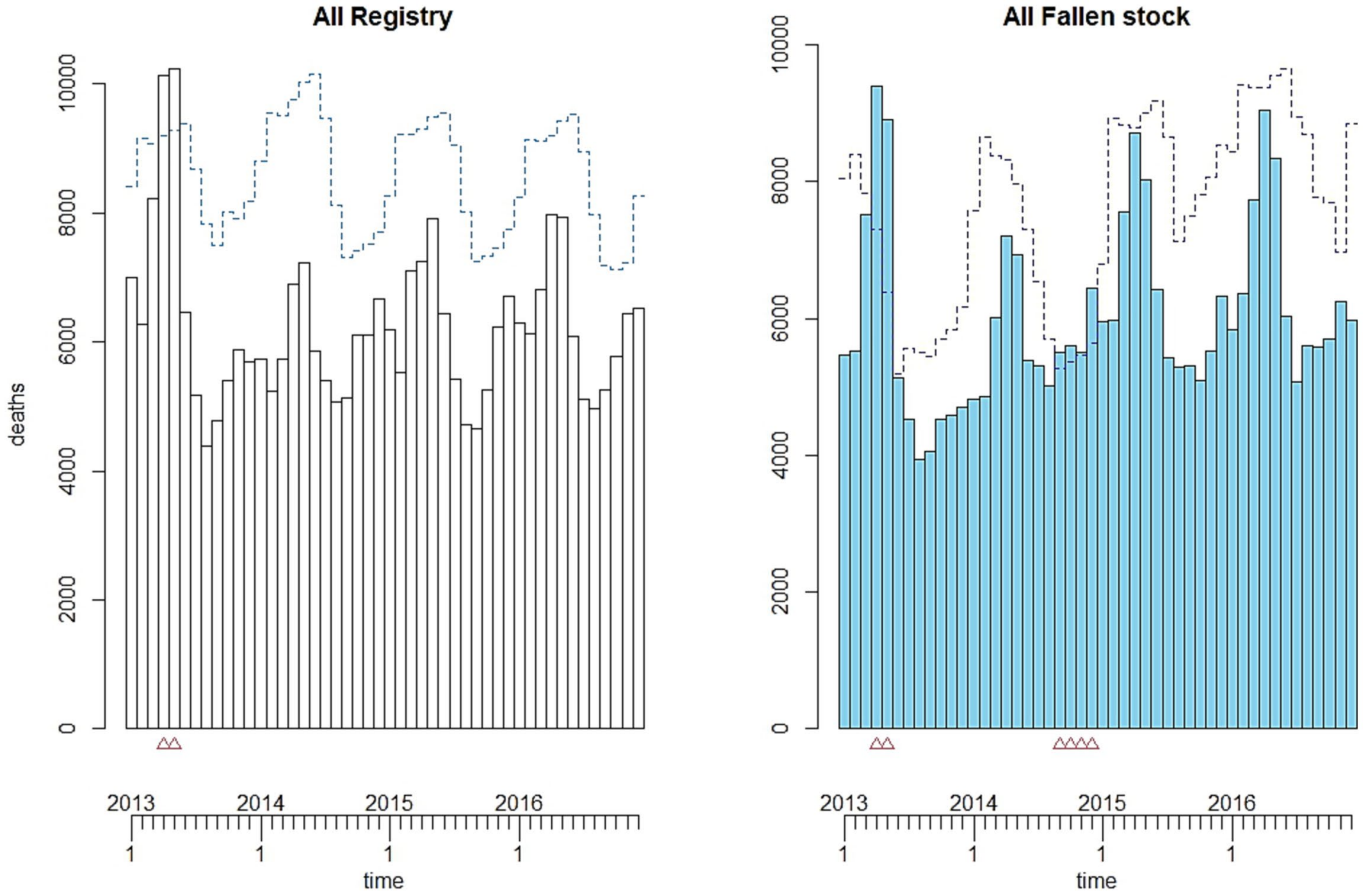
Monthly time series plots of number of all-age deaths (bar charts) and temporal aberration detection algorithm (TADA) (blue dotted lines) fitted to the two datasets. Alarms are indicated by the red triangles.

The potential utility of the two datasets for detection of population-level change was examined by fitting TADA to the monthly time series of each of the data series using the first 24 months as the historic reference period (Figure 5). The predicted upper bound of the fitted TADA is represented in Figure 5 by the blue dotted lines, the bars are the monthly observations. Aberrations (red triangles) are indicated whenever the observed (bars) are higher than the upper bound. Aberrations were detected in both dataset in April and May of 2013 as indicated by the alarms. Further alarms were highlighted in the fallen stock collections data from September to December of 2014.

**Figure 6:**
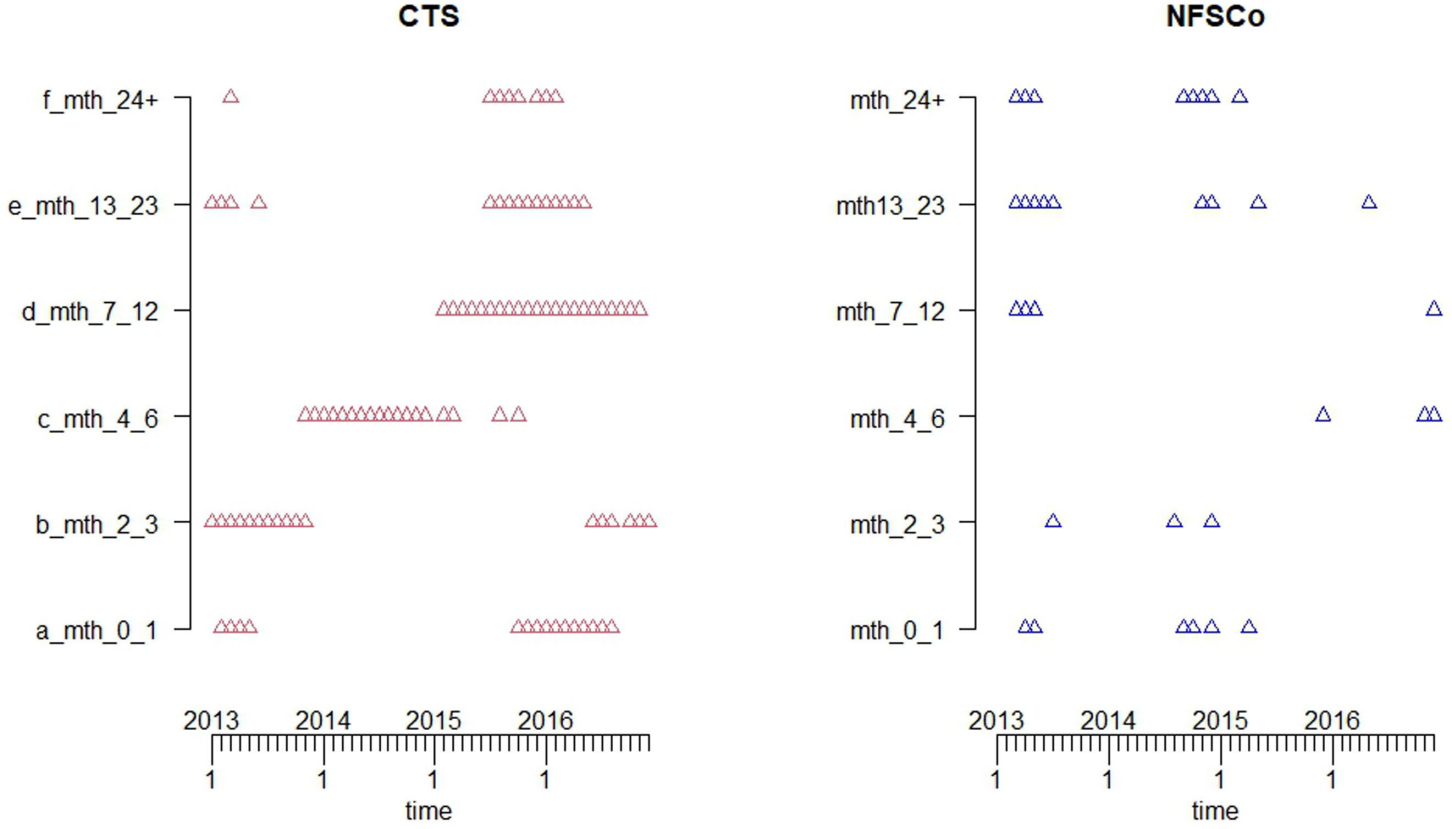
Alarms extracted from the temporal aberration detection algorithm (TADA) fitted to the monthly time series of each of the age groups in the two datasets. Alarms are indicated by the red triangles for the registry (CTS) data and blue triangles for the fallen stock collections (NFSCo) data.

Variations in the detected deviations from the expected norms are evident between the different age groups in the two datasets (Figure 6). On the whole, there seem to be more aberrations detected for each age group in the registry dataset compared to corresponding age groups in the collection dataset.

### 3.4 Spatial coverage

**Figure 7:**
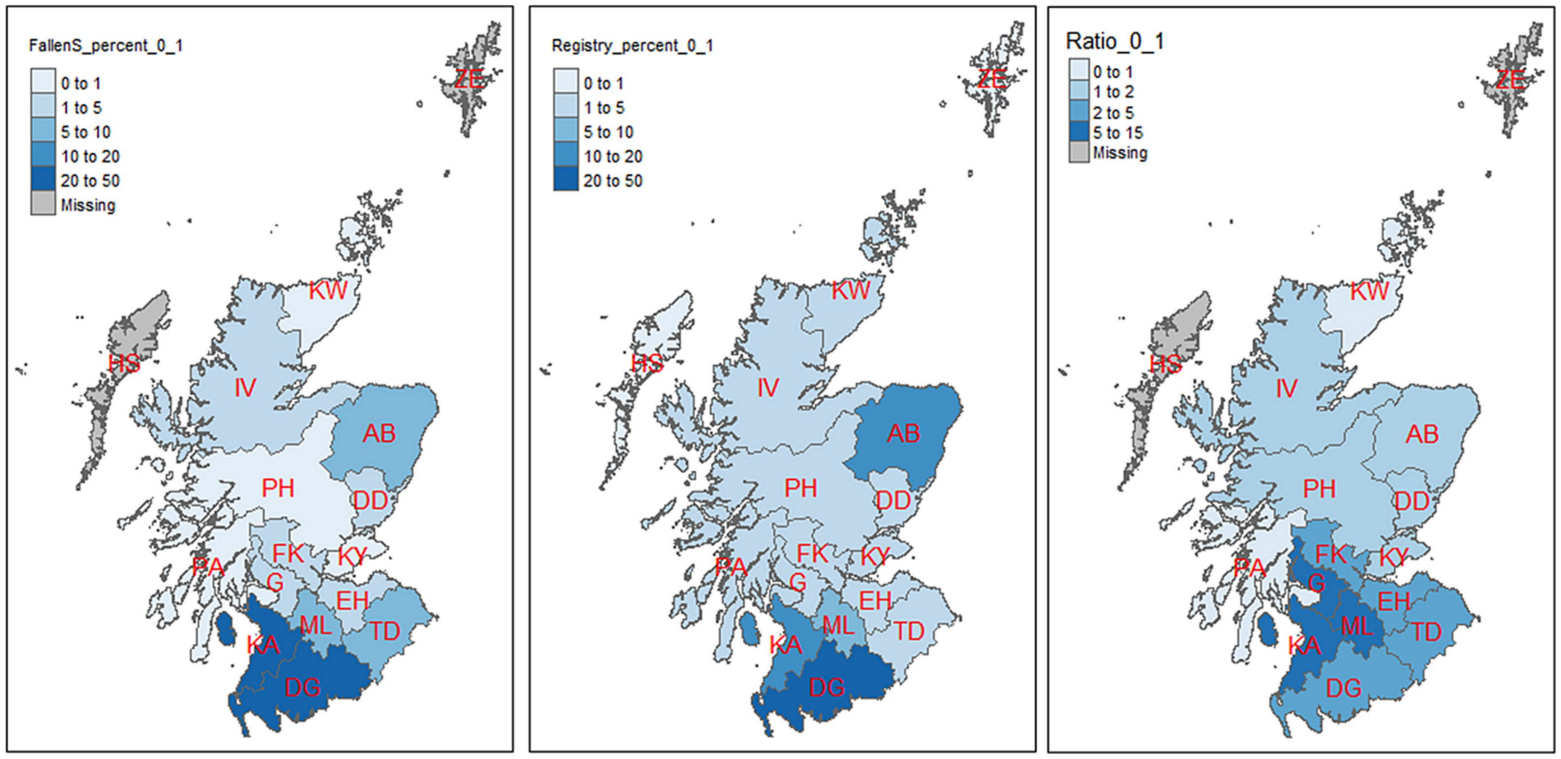
Percentage distribution of mortality in calves (age 0–1 month) by postcode area as represented by the two datasets (the first two maps). Third map is the ratio of 0–1 month old in the fallen stock collections (NFSCo) data to that of the registry (CTS) in each postcode area. There were no fallen stock collections (NFSCo) data from HS and ZE.

**Figure 8:**
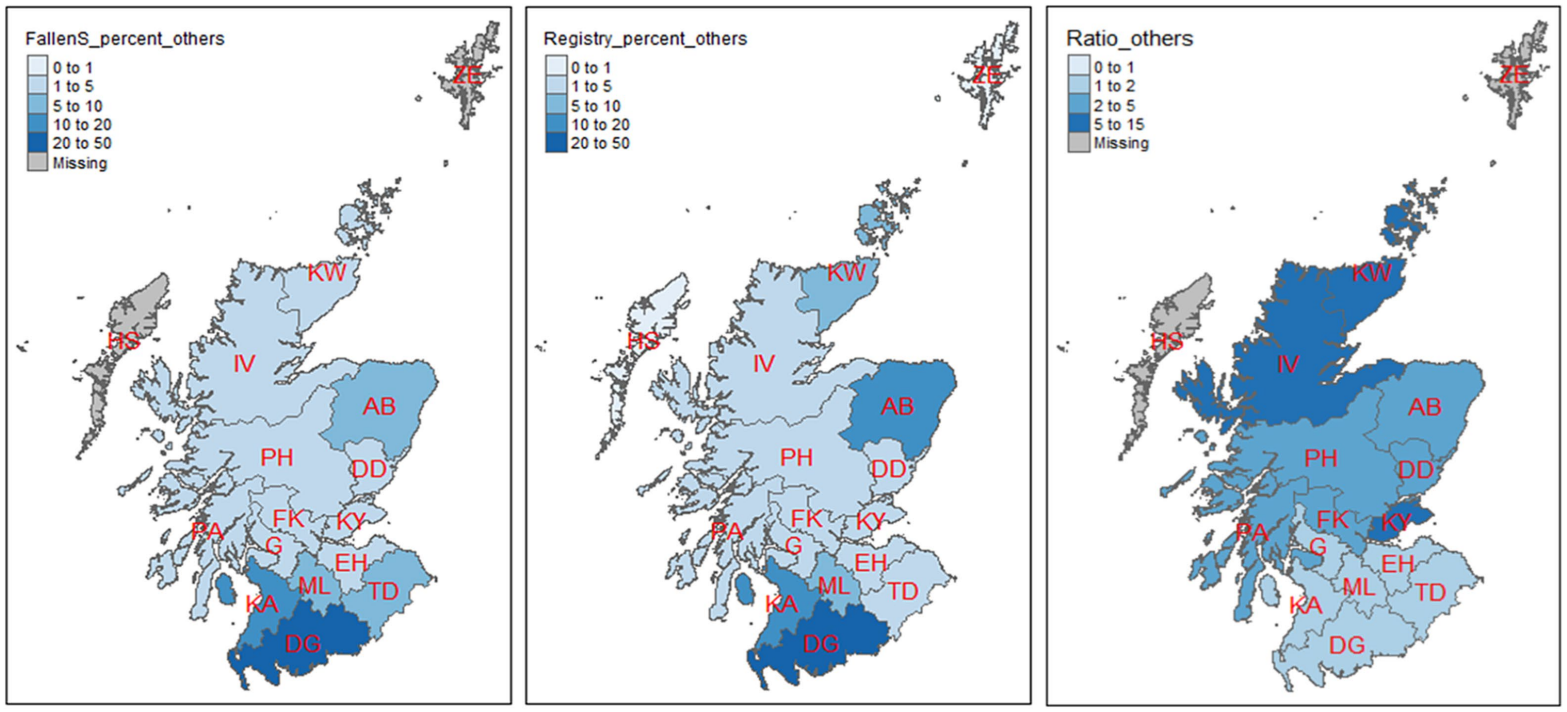
Percentage distribution of mortality in cattle aged 2 months and above by postcode area as represented by the two datasets (the first two maps). The third map is the ratio of cattle aged 2 months and above in the registry (CTS) data to that of fallen stock collections (NFSCo) in each postcode area. There were no fallen stock collections (NFSCo) data from HS and ZE.

The maps of the split data (0–1 month calves and the rest) are displayed in Figures 7, 8. The first and the second map in Figure 7 show the percentage of dead cattle collected and registered deaths by postcode area for calves aged 0–1 month for the fallen stock and the registry data, respectively. The distribution appears similar in both maps, with the south of Scotland [Dumfries & Galloway (DG)] accounting for about 46 and 36% of the total fallen stock and registered deaths, respectively. Kilmarnock (KA) followed, contributing 20 and 13%, then Aberdeen, with 5 and 12%, respectively. The third map represents the numbers in the fallen stock divided by that of registry data for calves aged 0–1 months. It shows how large the fallen stock collection data is relative to the registry data in each postcode. The number of 0–1 month calves collected as fallen stock in Motherwell (ML) and Kilmarnock (KA) was six times the number of deaths recorded in the registry data for these two postcode areas. Also, fallen stock collections of calves aged 0–1 month are five times the number of registered deaths in this age group in Glasgow (G) and Dumfries & Galloway (DG). More fallen stock aged 0–1 month were collected than were recorded in the registry data in 12 out of the 14 postcode areas that have both datasets.

The first and the second map of Figure 8 present the percentage of animals collected, or registered deaths, by postcode area for all data excluding calves aged 0–1 month for the fallen stock and the registry data, respectively. About 42% of the total collected as fallen stock in this age group were from Dumfries and Galloway (DG); only 27% of the total registered deaths, for this age group, were recorded in the registry data in the same area. The third map in Figure 8 displays the magnitude by which the registry data are greater than fallen stock collections in each postcode. More deaths were recorded in the registry data than were collected as fallen stock in all the 14 postcode areas that have both data. The registry recorded more than twice the numbers of fallen stock collected in 10 postcode areas. In Kirkwall (KW), the registry recorded more than 10 times the number of deaths than the number of fallen stock collected.

Overall, the registry data provide more comprehensive coverage of all postcode areas of Scotland. There were no fallen stock data from Outer Hebrides (HS) and Shetland (ZE) over the 6 years of study. There seems to have been poor coverage of fallen stock collections in the north of Scotland but there was a strong presence in central Scotland.

**Figure 9:**
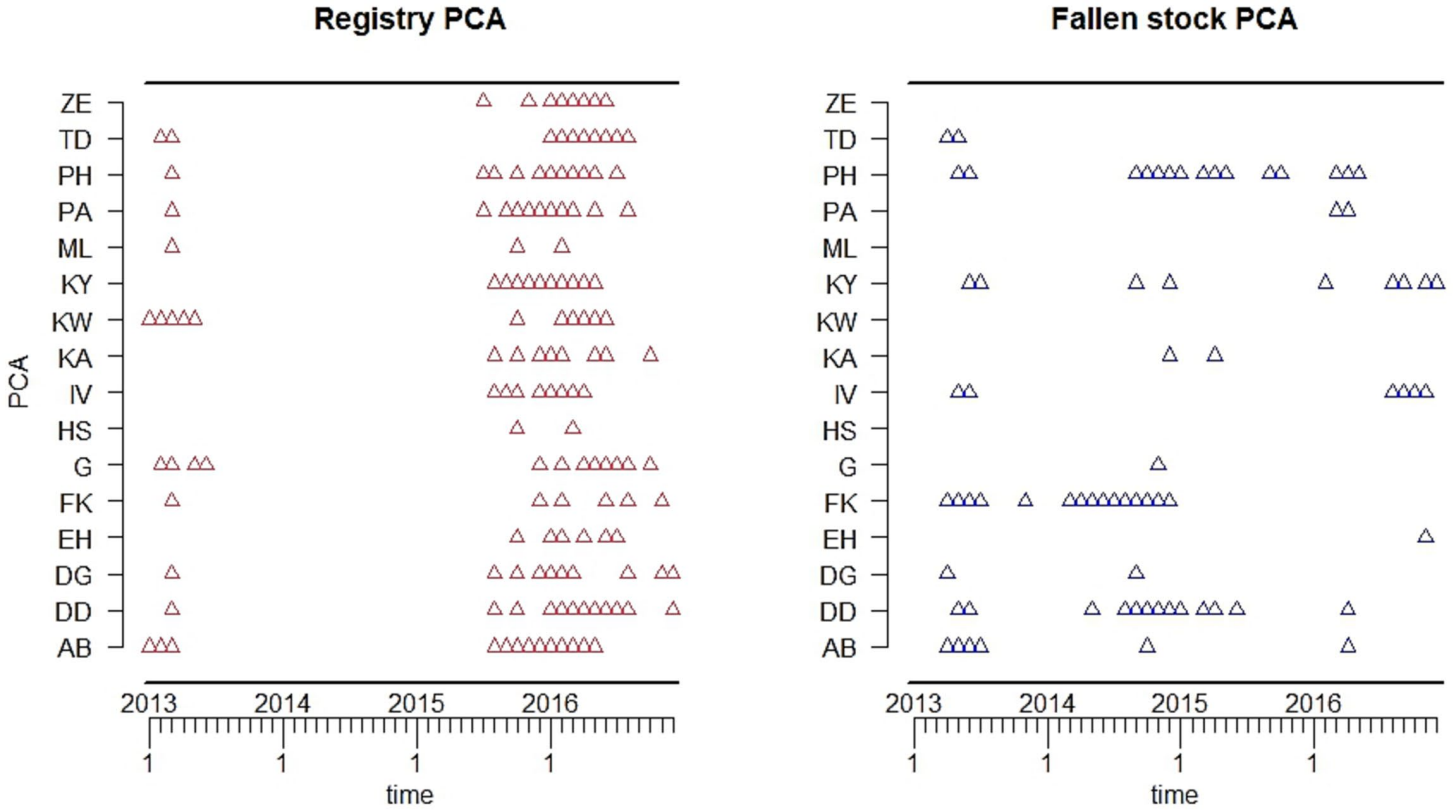
Alarms extracted from the temporal aberration detection algorithm (TADA) fitted to the monthly time series of each postcode area in the two datasets. Note: The were no fallen stock collections (NFSCo) data from HS and ZE.

The outputs of the temporal analyses of the deviations from expected mortality show differences in the timepoints indicated as alarm points, between the datasets in each Scottish postcode area (Figure 9). Overall, there are more aberrations in the registry data. However, aberrations were detected in 2013 in both datasets with some points coinciding in the two datasets.

## 4 Discussion

We have retrospectively analyzed the mortality experienced by the Scottish cattle population between 2011 and 2016 as provided by two different data sources namely, non-statutory collections by the National Fallen Stock Company cattle data and the mandatory death records, or registry, of the Cattle Tracing System managed by the British Cattle Management Services. Data from these two sources were compared with a view to establish their strengths and limitations in relation to their potential use in animal health surveillance as proxies for mortality for the Scottish cattle population. This was achieved by comparing the spatial and temporal patterns and other characteristics of fallen stock collections with that of the statutory dataset and their subpopulations. We have found that while there are some similarities in the information provided by each dataset, there are also some important differences. These findings have implications for their utility for the provision of surveillance intelligence.

As a statutory register, the registry data should provide the most comprehensive coverage of the mortality experienced by the cattle population, as all deaths should be notified to the central register within 7 days. This appears to be the case, with the aggregate number of deaths recorded over the period of study slightly (1.2%) higher compared to the aggregate fallen stock collections within the same period. However, the overall difference was smaller than expected given knowledge of NFSCo membership during this period (pers.comm. NFSCo).

### 4.1 Age effects

When the datasets were stratified by age groups, a different picture emerged. The analyses indicate that the fallen stock collections data provide a more comprehensive measure of the mortality experience in 0–1-month-old calves, with collections consistently about 3.8 times the number of deaths recorded in the registry data for this age group, in each month. The fallen stock collections data probably provides a more accurate representation of the mortality experienced on-farm by this age-group than the registry data does. This is most likely to be because farmers are not required to report a calf’s death to BCMS if it dies prior to ear-tagging and registration. In Scotland, farmers must fit ear tags before a beef calf is 21 days old, or when it moves off the holding if that is earlier; dairy calves must have a primary ear-tag by 36 h after birth, and for both beef and dairy calves, they must be registered within 27 days after birth (20). If the calf dies before these deadlines, then they do not need to be registered centrally and electronically, only locally on-farm in the holding register. Consequently, all perinatal and most neonatal deaths will not be recorded in the registry data. By definition (see Introduction), these collections may also include still-born animals that would never be registered. However, the carcases will be collected for disposal by fallen stock services, such as NFSCo. This finding has implications for further interpretation of outputs from these data in this study and it also highlights the need to consider this data-gap carefully when conclusions are drawn, for this age-group, from other analyses of the registry data [e.g., (3, 5, 21)]. Whether this is a factor specific to the British datasets, or more widely relevant, requires further investigation. In a comparison of the utility of fallen stock and national cattle register data in France, Perrin et al. (7) noted that there was a potential difference between the number of cattle collected for disposal and deaths notified, which needed further investigation; in that study the former was higher than the latter. However, they did not publish age-specific details. It may well be possible that a contributory factor to their higher disposal figures is mortality in young calves, as identified in our study. The data-gap is also of relevance for population, livestock industry, or farm-level estimation of the impact of early calf mortality on green-house gas (GHG) emissions. With the move to achieve Net Zero and changes to agricultural policies, such calculations are being made for aspects of animal health such as liver fluke in cattle (22, 23). If the impact of early calf mortality is calculated using registry data, or successor registers (e.g., Scotmoves+ and the Livestock Information Service) they will be under-estimates.

Once cattle are registered, the registry data should provide comprehensive coverage of mortality experienced in the population. Hyde et al. (3) studied calf mortality between 2011 and 2018 in Great Britain and found that 25% of on-farm deaths happened within the first 3 months of age, with 54% occurring before 24 months of age. This is similar to our finding for Scotland only, which is as expected given no regional variations as we were using both a spatial and temporal subset of the same data source.

### 4.2 Coverage and spatial effects

There were twice as many deaths recorded in the registry data compared to fallen stock collected in each age group over 1 month of age in the study period. This is different to the aforementioned direction of the difference observed in preliminary analysis by Perrin et al. (7). In their French study, the fallen stock data were obtained from an interface where data from all the major collection services were centralized. In Britain, there is, as yet no similar centralized dataset. While there is a statutory requirement to arrange appropriate disposal of a bovine animal that dies on farm, there is no requirement to use the NFSCo service. NFSCo’s coverage is determined by its membership and the geographical spread of the members. There are other collection services across Scotland, as well as Scottish cattle producers who are not NFSCo members; information about the numbers of carcasses collected by non-member providers, from non-member producers, are effectively lost for surveillance purposes.

This effect of NFSCo membership has to be considered when interpreting the geographic distribution of these fallen stock collections data. They suggest poor coverage in the north of Scotland, Anecdotally, the availability of alternative fallen stock collection services may be part of the reason behind the registry/ collected ratios that are more than 1 in some of these postcode areas. Another contributory factor in some postcode areas, especially those with lower livestock densities and in the crofting community areas is the ‘remote area’ derogation. This facilitates on-farm disposal and it applies in a number of areas in the north-west, Highlands and Islands (IV, KW, KY, HS, ZE). In conjunction with the exemption from TSE testing in the Scottish islands for animals over 48 months, it may lead to reduced availability of collection services and thus to less reported fatalities via fallen stock collections. There are, however, some postcode areas [Dumfries & Galloway (DG), Glasgow (G), Kilmarnock (KA), Motherwell (ML)] where fallen stock were collected than were recorded deaths in registry. Further examination of the data reveal that, as previously discussed, this was an age-related effect. In these postcode areas, far more collections of calves aged 0–1 month were made than in the other age groups. These areas have both high-density cattle population and are relatively easily accessible for collection and transportation. It is most likely that—as previously discussed—these calves died before they were registered, or even tagged, thereby not making it into the registry records. The early stages of life are known to be the most hazardous with higher levels of mortality noted in other studies (4, 24, 25). Findings from a systematic review highlight that approximately 80% of all mortalities, which occur in cattle within the first 12 months, had occurred by 6 months of age (26), with the majority of calves dying within the first month (27, 28).

### 4.3 Seasonal influences

Broadly, the overall all-age seasonal and trend patterns were similar in the two datasets, both depicting a seasonal peak in the spring, trough in the summer and increased mortality again in the late autumn and early winter. Several authors (3, 7, 29, 30) have reported similar seasonal variations in cattle mortality. The patterns were different for each of the age groups over 12 months of age and the size of seasonal effects was also age-dependent, varying between 1.4 and 2.0 times the yearly average. These variations in mortality reflect the seasonal patterns expected from knowledge of the British cattle production-year calendar and may reflect the association between mortality and the production cycle. For example, the aforementioned early age calf mortality, deaths due to calving injuries in heifers and cows combined with the predominance of Spring calving, and seasonally-associated disease influences such as spring turn-out, liver fluke, as well as environmental factors such as those described by Hyde et al. (3). Another contributory factor to this effect might be a result of looking at count data; the peaks of mortality in the 0–1 month age-group in April/May, bounded by March and June reflect the increased age-specific denominator population due to the predominance of the spring, rather than autumn, calving season in Scotland (21) and the larger Scottish National Beef Herd, rather than year-round or batch calving Dairy Herd. This age-specific denominator population effect then progresses through the age-groups with the peak in mortality for the 2–3 months age-group being May/June/July and so on. While it is possible to describe a denominator population and estimate risks and rates, as has been done before with the registry data (3, 27), this was not possible with the fallen stock collections data. This is because of the format in which the data was available. From experience with the equivalent sheep fallen stock collections data (31), even if this could be addressed, an appropriate denominator would be difficult to determine.

### 4.4 Count data, alerts, and other data limitations

To be able to make comparisons between the two datasets and to see whether they give us similar, or dissimilar surveillance information, we were, therefore, restricted to the application of statistical methods to detect deviations from the expected norms on mortality counts. Both datasets were amenable, with the position of alarms indicating points of deviation from expected deaths in the spring of 2013. These were similar in both all-age datasets. The spring of 2013 was particularly harsh with severe blizzards in many areas of Scotland during the latter part of March with consequent livestock fatalities (32).

Additional alert points were indicated in the fallen stock collections dataset in autumn of 2014, for which the cause is not clear. From the stratified analyses it would appear that these occurred mostly in post-code areas in the high cattle density south-west of Scotland, in calves up to 3 months of age and adults (over 13 months of age). This might suggest something associated with the autumn calving period. Similar alarms are not raised in the registry data, either overall (all-age), or in the spatially stratified analysis. There are alarms at that time in only the youngstock age-groups, which are not seen in the fallen stock collections analyses (4–12 months). This is just one example of differences in variation in detected deviations from expected norms in the two datasets, with more alarms indicated in the registry dataset compared to the corresponding age group in the fallen stock collections. The age-specific, geographic coverage, and membership effects discussed earlier are reflected in the distribution of detected TADA alarms across the postcode areas and have to be considered when attempting to interpret these alarms. In addition, this may be a reflection of the trend patterns seen in the fallen stock collections age groups. These seem to have higher mortality in the first 24 months of the study period, relative to 2013–2014. This period (2011–2012) was used as the historical reference period. This may be due to a decline in the number of members over time. Another explanation could be due to the fact that the registry dataset had better coverage of the older age groups. There is also the potential for a degree of age misclassification to be considered in the fallen stock collections data, as this is farmer reported, rather than calculated as in the registry data.

### 4.5 From applied research to operational information production

We have demonstrated that both the Scottish cattle registry and the fallen stock data can be analyzed retrospectively, and that while they tell similar stories, there are important differences, The most important difference is that the fallen stock collections data captures early calf more accurately than the registry data. If there are changes in the mortality experienced in the up to 1 month old group then they are less likely to be detected in the registry dataset. However, given the uneven geographical coverage of the collections, there is a potential for bias in the outputs from the fallen stock collections data, if the cause of the mortality experienced in the over 1 month age-group is spatially associated. Other important differences include the more complete coverage of cattle over 1 month of age through the registry data and the fact that the registry data have the advantage that they can be further stratified, for example, by type of production (dairy/beef) or breed, which may be of further interest. Finally, it is possible to identify both individual animals and their farms in the registry data, which is not possible in the fallen stock data. Therefore, the two datasets cannot be matched and there is the possibility to define a denominator and look at mortality rates with the registry data. Given that each data source has its strengths and weaknesses, parallel analysis of both would be complementary and ideal for operational purposes. Alternatively, advanced surveillance statistical approaches could be applied to jointly model both datasets and produce estimates that combine the strengths in both datasets.

To further develop animal health surveillance, periodic retrospective analysis could be automated and implemented providing data could be accessed in a timely manner and technological issues were not an obstruction. This has been achieved elsewhere (11). It would require further investment of resources as well as policy decisions about the time-scale of the surveillance and the purpose of the analysis. Within this study, the time-scale used was a month, as this was the level of aggregation that the fallen stock collections data are produced (for invoice and billing purposes) and provided in. The cattle registry data has the potential to be utilized at a finer scale; however, that brings with it various challenges such as timely access and determining the most appropriate surveillance scale as discussed by Perrin et al. (7). A change in legislation in Scotland, effective from October 2021, mandated that all Scottish cattle farmers in the country should report all cattle deaths to Scotmoves+. ScotMoves+ is managed by ScotEID, a multispecies database that also handles sheep, goat and pig movements in Scotland, and will act as the electronic holding register for all Scottish cattle keepers (20). At present the current Scottish cattle registry, Scotmoves+, data is available retrospectively on a more regular basis to the EPIC consortium. Its use for operational purposes is being further investigated. In addition, the provision of expertise, with industry knowledge, to not only interpret the outputs but also to investigate and follow-up alerts and alarms once they have been produced from the data, will need to be resourced. This may be achieved in Scotland through the proposed Veterinary Surveillance Intelligence Unit (VSIU).

The question raised by Perrin et al. (7) as to whether mortality monitoring provides any advantage over and above existing surveillance systems, remains a moot point. This, plus the discussions of whether, or not, monitoring mortality is a timely way to detect unexpected events and whether it is appropriate to call it syndromic surveillance, or not, probably contribute to the chasm that exists between investigations of the utility of data-sources as potential contributors to surveillance and advancement into operational systems. The steps that need be taken to build bridges and span this chasm are about more than basic data availability and appropriate technological requirements, although these are basic essentials. Often, the ability to advance animal health surveillance is hampered by the conundrum that until it can be demonstrated to be a worthwhile investment of resources, the resources required cannot be identified. Sometimes it requires a leap of faith; other times, a slow build of momentum and the perseverance to build sufficient evidence to progress is being made, able to make it to a tipping point. This study provides a foundation on which to build evidence contributing to further development of an operational system.

Further investigations using these data sources have occurred since this study was completed. They include: a pilot to see if the fallen stock collections data could be enhanced by collecting the “farmer-reported cause of death,” in broadly defined categories, at the time of collection (pers. comm NFSCo); more detailed examination of the structure and efficiency of the Scottish Beef Cattle Herd (21); development of some of the algorithms developed for data-handling in Thomson et al. (21) into a potential farm-level benchmarking system and their application along with additional statistical aberration detection mechanisms to the registry data (ongoing within the EPIC consortium). Progress is being made; all be it slowly.

## 5 Conclusion

In Great Britain, there are two data sources routinely collected for primary purposes other than surveillance that contain data associated with mortality in cattle. These are the statutory registration of births, deaths and movements and fallen stock collections for disposal through voluntary membership scheme. We have produced the first retrospective analysis of the Scottish fallen stock collections dataset for cattle, in terms of frequency, space and time for 2011–2016. From these data, we can conclude that there is a data-gap in the mortality in calves up to 1 month of age recorded in the registry data. Therefore, early calf mortality estimates made using CTS data, or successor registers (such as Scotmoves+ and the Livestock Information Service) will be under-estimates. The retrospective analyses we conducted demonstrated that the two data sources can be utilized for monitoring the mortality experienced by the Scottish cattle herd. They are positively correlated and provide broadly similar information about seasonal and trend patterns, but some differences exist in their demographic and spatial coverage. However, they each have strengths and weaknesses, so ideally, a system where they are analyzed and interpreted in parallel or jointly would be the best way to optimize the information obtained for surveillance purposes for epidemiologists, risk managers, animal health policy-makers and the wider livestock industry sector.

## Data availability statement

The data analyzed in this study is subject to the following licenses/restrictions: The data were obtained from third parties: the National Fallen Stock Company and British Cattle Management Services (BCMS). Request for access to any of these data should made directly to respective organization. Requests to access these datasets should be directed to https://nfsco.co.uk/; https://www.gov.uk/government/organisations/british-cattle-movement-service.

## Author contributions

JE: Conceptualization, Data curation, Formal analysis, Investigation, Methodology, Software, Visualization, Writing – original draft, Writing – review & editing. CC-G: Funding acquisition, Validation, Supervision, Writing – review & editing. GG: Validation, Writing – review & editing. ST: Conceptualization, Data curation, Validation, Writing – original draft, Writing – review & editing.

## Footnotes

^1^The EPIC data repository is a centrally curated collection of data resources established in 2011. The datasets are provided to the Scottish Government’s Centre of Expertise on Animal Disease Outbreaks (EPIC) by individual agencies or institutions (the data-providers). Permissions for access and use for individual studies are granted, by arrangement with the data-providers, to support research within the Scottish Government’s Strategic Research Programme.

## Supplementary material

SUPPLEMENTARY FIGURE S2 Monthly time series plots of mortality for over 12 months old by data sources. The smooth trend is shown in red.

SUPPLEMENTARY FIGURE S2A Average seasonal effects by age groups. Monthly effect is different depending on the age group. The green line represents average mortality each year.

SUPPLEMENTARY FIGURE S2B Seasonal patterns of mortality for over 12 months old, by the two data sources. Fairly similar seasonal patterns per age group but monthly variation was higher in the fallen stock collections data.

SUPPLEMENTARY FIGURE S3 Scatter plot of the monthly time series of registry (CTS) and fallen stock collections (NFSCo) cattle mortality data for all-age groups. Strong positive correlation exists between the monthly time series of the two datasets.

SUPPLEMENTARY FIGURE S4 Scatter plots of the monthly time series of registry (CTS) and fallen stock collections (NFSCo) cattle mortality data by age groups over 12 months old. Positive correlation exists between the monthly time series of the age groups in two datasets.
