# Supplementary material for: Bovine mortality: the utility of two data sources for the provision of population-level surveillance intelligence
Source: Front Vet Sci. 2024 Feb 7;11:1270329. doi: 10.3389/fvets.2024.1270329 (PMC10880450; doi:10.3389/fvets.2024.1270329)
Supplement: Supplementary file 1 [file Data_Sheet_1.docx]

Dairy Cattle | Helping farmers in Scotland | Farm Advisory Service (fas.scot).

**A brief introduction to the Scottish cattle population**

The Scottish cattle population includes a diverse dairy industry and a beef industry, both with multiple production and management systems The distribution of these industries is driven by geography, weather and land-use suitability. For example: much of the north and west of the Highlands and Islands consists of remote rural, crofting, communities. Most of the commercial Scottish dairy herds are now located in the south west of Scotland Scottish Dairy Herd Analysis 1st. January 2022 - Press.pdf (thesdca.co.uk), which with the north-east are the two regions that have the highest density of cattle. The number of dairy herds has declined by 195 over the last 10 years to a total of 832. Meanwhile the average dairy herd size has risen JACK LAWSON (thesdca.co.uk). As stated in Thomson et al, 2020, “the structure of Scotland’s cattle systems is complex with both dairy and suckler herds feeding into specialist finishers who in turn largely manage the supply of finished ‘prime’ beef production.” As with the dairy sector, there has been a decline in the total beef breeding herd over the past decade Livestock - Results from the Scottish Agricultural Census: June 2021 - gov.scot (www.gov.scot). While the dairy herds are predominantly calve all year round Dairy Cattle | Helping farmers in Scotland | Farm Advisory Service (fas.scot), calving amongst the suckler beef herd is concentrated in the spring (Thomson et al, 2020).
