# Supplementary figures and images for: Bovine mortality: the utility of two data sources for the provision of population-level surveillance intelligence
Source: Front Vet Sci. 2024 Feb 7;11:1270329. doi: 10.3389/fvets.2024.1270329 (PMC10880450; doi:10.3389/fvets.2024.1270329)

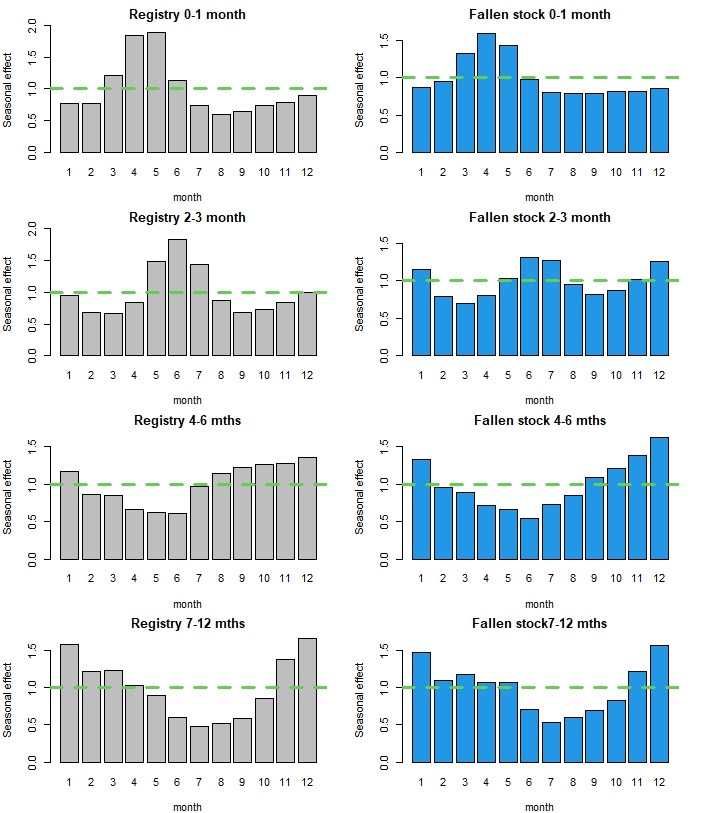

Supplement: SUPPLEMENTARY FIGURE S2 — Monthly time series plots of mortality for over 12 months old by data sources. The smooth trend is shown in red. [file Image_1.JPEG]

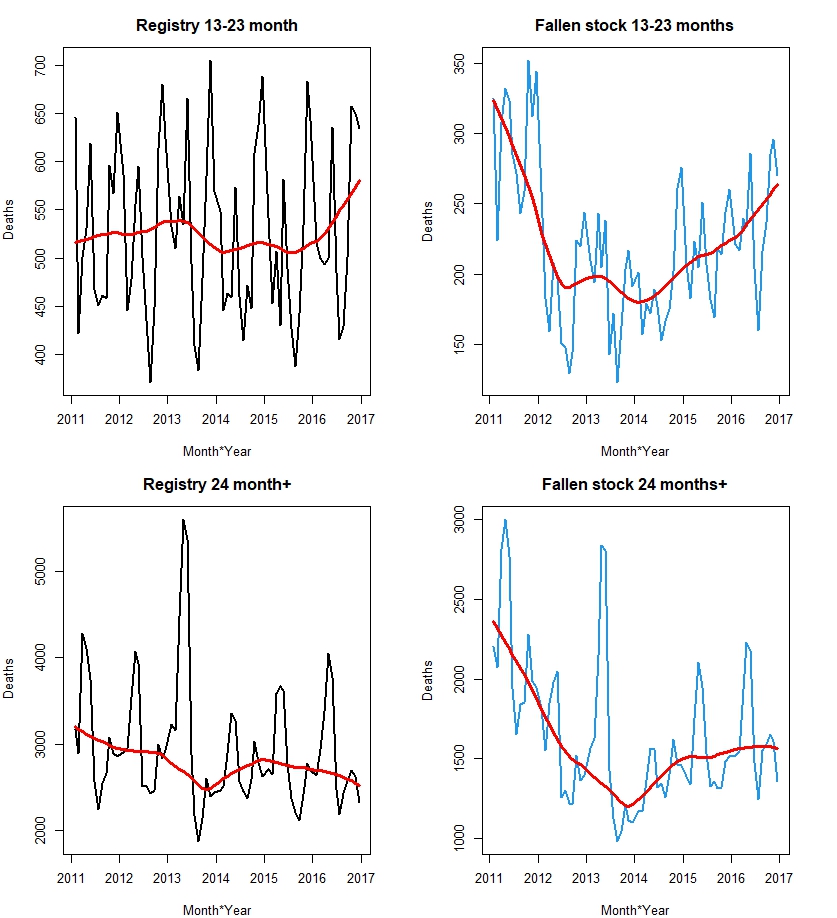

Supplement: SUPPLEMENTARY FIGURE S2A — Average seasonal effects by age groups. Monthly effect is different depending on the age group. The green line represents average mortality each year. [file Image_2.JPEG]

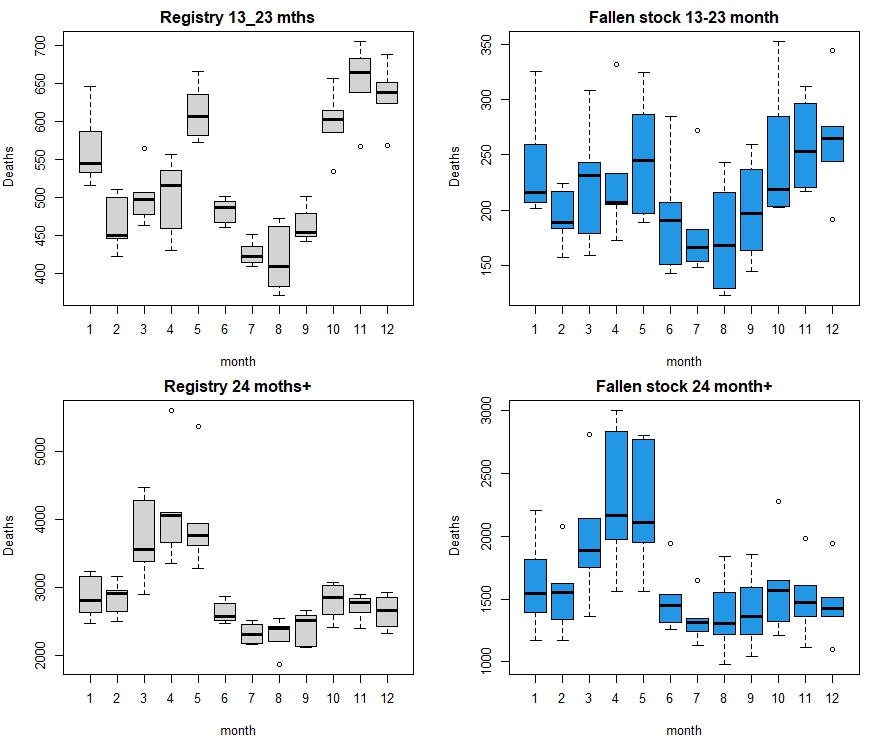

Supplement: SUPPLEMENTARY FIGURE S2B — Seasonal patterns of mortality for over 12 months old, by the two data sources. Fairly similar seasonal patterns per age group but monthly variation was higher in the fallen stock collections data. [file Image_3.JPEG]

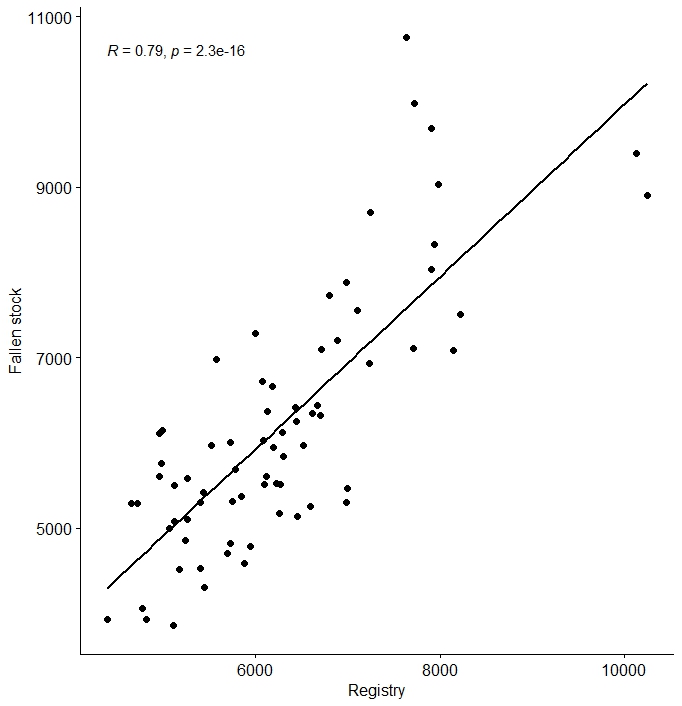

Supplement: SUPPLEMENTARY FIGURE S3 — Scatter plot of the monthly time series of registry (CTS) and fallen stock collections (NFSCo) cattle mortality data for all-age groups. Strong positive correlation exists between the monthly time series of the two datasets. [file Image_4.JPEG]

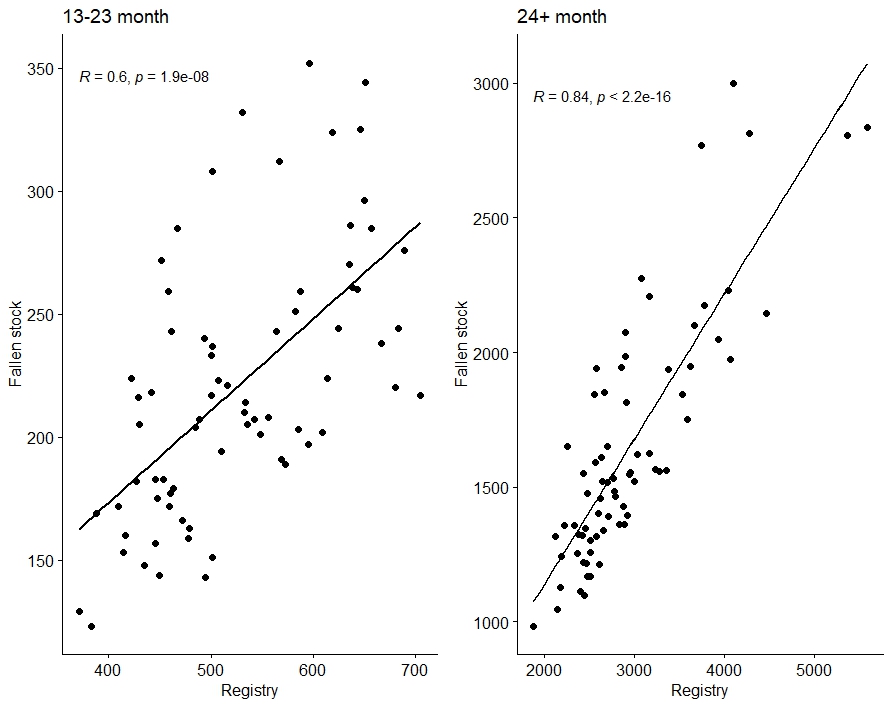

Supplement: SUPPLEMENTARY FIGURE S4 — Scatter plots of the monthly time series of registry (CTS) and fallen stock collections (NFSCo) cattle mortality data by age groups over 12 months old. Positive correlation exists between the monthly time series of the age groups in two datasets. [file Image_5.JPEG]
